# Supplementary material for: MetaRibo-Seq measures translation in microbiomes
Source: Nat Commun. 2020 Jun 29;11:3268. doi: 10.1038/s41467-020-17081-z (PMC7324362; doi:10.1038/s41467-020-17081-z)
Supplement: Supplementary file 10 — Supplementary Data 7 [file 41467_2020_17081_MOESM10_ESM.zip › File2/Confidence_High_Taxonomy/320246_out.krona.html]

Javascript must be enabled to view this page.

members
magnitude
magnitudeUnassigned
count
unassigned
taxon
rank

320246\_out

2
109

SRS020628\_contig\_number\_25910SRS1041181\_contig\_number\_9674

1
2

SRS062544\_contig\_number\_24286
superkingdom
105

phylum
83
976

class
200643
83

order
83
171549

family
8
815

genus
8
816

species
8

SRS015797\_contig\_number\_21480SRS023926\_contig\_number\_34007SRS065299\_contig\_number\_contig-100\_117.118SRS077308\_contig\_number\_11901SRS077916\_contig\_number\_10701SRS078425\_contig\_number\_21797SRS098704\_contig\_number\_contig-100\_548.549SRS143062\_contig\_number\_12031
1262741

4
171552

SRS015985\_contig\_number\_contig-100\_1011.51371SRS018875\_contig\_number\_29670SRS049147\_contig\_number\_21136SRS104711\_contig\_number\_4168
75
family

1283313
3
genus

species
76122

SRS019488\_contig\_number\_5482
1

712469
2

SRS104962\_contig\_number\_6776SRS143231\_contig\_number\_4888
species

species
2

SRS015590\_contig\_number\_15089SRS101439\_contig\_number\_15950
1852368

species

SRS012279\_contig\_number\_18284SRS014689\_contig\_number\_contig-100\_83.59100SRS016133\_contig\_number\_16891SRS018055\_contig\_number\_contig-100\_706.74907SRS019126\_contig\_number\_18679SRS019792\_contig\_number\_4557SRS021897\_contig\_number\_14413SRS022464\_contig\_number\_5444SRS045127\_contig\_number\_15531SRS053854\_contig\_number\_19790SRS054976\_contig\_number\_22756SRS056327\_contig\_number\_10939SRS056896\_contig\_number\_14233SRS057791\_contig\_number\_35558SRS062878\_contig\_number\_contig-100\_108.138508SRS075813\_contig\_number\_9993SRS075987\_contig\_number\_17552SRS077384\_contig\_number\_4790SRS098065\_contig\_number\_9100SRS098612\_contig\_number\_13716SRS098620\_contig\_number\_10628SRS104165\_contig\_number\_41672SRS104314\_contig\_number\_19581SRS142545\_contig\_number\_1181SRS143037\_contig\_number\_24161SRS143354\_contig\_number\_10898SRS143423\_contig\_number\_24317SRS143879\_contig\_number\_22584SRS146738\_contig\_number\_14181SRS147126\_contig\_number\_24664SRS147149\_contig\_number\_21548SRS147226\_contig\_number\_27974SRS147380\_contig\_number\_26708SRS147980\_contig\_number\_6392SRS148973\_contig\_number\_16881SRS149671\_contig\_number\_28085
36
370804

4

SRS056896\_contig\_number\_13477SRS144300\_contig\_number\_33139SRS146894\_contig\_number\_35666SRS893350\_contig\_number\_31379
838
30
genus

species
1

SRS148303\_contig\_number\_3576
28132

1262935

SRS049389\_contig\_number\_30709SRS103971\_contig\_number\_14003SRS148284\_contig\_number\_32445
3
species


SRS097912\_contig\_number\_16673SRS143088\_contig\_number\_2875
2
1947120
species

species

SRS021528\_contig\_number\_14925
1
425941

species

SRS018923\_contig\_number\_4173SRS053603\_contig\_number\_23512SRS1055063\_contig\_number\_18583SRS147094\_contig\_number\_20243
4
159272

species

SRS045602\_contig\_number\_3715
1
1433844

species
1

SRS104647\_contig\_number\_7392
470565

species

SRS018679\_contig\_number\_16126SRS078182\_contig\_number\_32146SRS143450\_contig\_number\_8516
3
1262937

228604
2

SRS017808\_contig\_number\_24712SRS144090\_contig\_number\_11363
species

60133
3

SRS019696\_contig\_number\_16394SRS076764\_contig\_number\_22731SRS077092\_contig\_number\_20675
species

1947201

SRS018439\_contig\_number\_7097SRS893348\_contig\_number\_18903SRS893359\_contig\_number\_16401
3
species

species
1

SRS057244\_contig\_number\_15335
228603


SRS149720\_contig\_number\_8417
1
2079531
species

phylum
1224
12

class
10
1236

order
72274
10

10
468
family

469
10
genus

10
909768
species group

species

SRS016740\_contig\_number\_11536SRS104830\_contig\_number\_21148SRS142680\_contig\_number\_25316SRS143088\_contig\_number\_14459SRS144194\_contig\_number\_18362SRS144376\_contig\_number\_6208SRS144609\_contig\_number\_20199SRS144644\_contig\_number\_14933SRS147045\_contig\_number\_7544SRS893324\_contig\_number\_11390
10
470

class
2
28216

2
206351
order

2
481
family

482

SRS147712\_contig\_number\_16599
1
2
genus

species
1

SRS148150\_contig\_number\_21184
1715112

phylum
9
1817803

1817863
9

SRS013705\_contig\_number\_contig-100\_968.206945SRS014470\_contig\_number\_36892SRS014684\_contig\_number\_26702SRS042643\_contig\_number\_31997SRS048791\_contig\_number\_13033SRS103971\_contig\_number\_31813SRS104087\_contig\_number\_contig-100\_1026.124354SRS105068\_contig\_number\_27656SRS143604\_contig\_number\_contig-100\_1571.169085
species

2

SRS047824\_contig\_number\_30410SRS075765\_contig\_number\_contig-100\_1145.111531
